# Translational Research in Audiology: Presence in the Literature

**DOI:** 10.3390/audiolres12060064

**Published:** 2022-11-28

**Authors:** Agnieszka J. Szczepek, Ewa Domarecka, Heidi Olze

**Affiliations:** 1 Department of Otorhinolaryngology, Head and Neck Surgery, Charité–Universitätsmedizin Berlin, 10117 Berlin, Germany; 2 Faculty of Medicine and Health Sciences, University of Zielona Gora, 65-046 Zielona Gora, Poland

**Keywords:** audiology, translational research, translational science, translational audiology

## Abstract

Translational research is a process that focuses on advancing basic research-based clinical solutions and is characterized by a structured process accelerating the implementation of scientific discoveries in healthcare. Translational research originated in oncology but has spread to other disciplines in recent decades. A translational project may refer to pharmacological research, the development of non-pharmacological therapies, or to disease monitoring processes. Its stages are divided into basic research focused on the clinical problem (T0), testing the developed means in humans (T1), conducting trials with patients (T2), implementation and dissemination of successful approaches (T3), and improving community health (T4). Many audiological studies are translational in nature. Accordingly, this scoping review aimed to evaluate the use of the terms “translational audiology” and “translational research in audiology” in the literature and examine the goals of the identified studies. PubMed and Web of Science search identified only two publications meeting the search criteria. We conclude that identifying translational audiological studies in the literature may be hampered by the lack of use of the terms “translational audiology” or “translational research”. We suggest using these terms when describing translational work in audiology, with a view to facilitating the identification of this type of research and credit it appropriately.

## 1. Introduction

**Figure 1:**
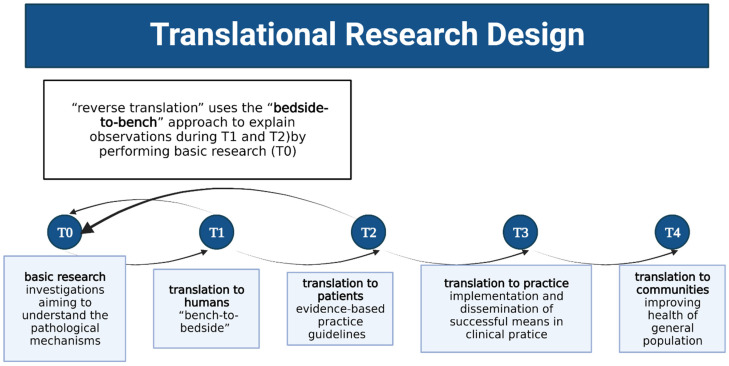
Types of translational research based on the description by Wichman et al. (2021) [2]. Created with BioRender.com.

Translational research (TR) is a biomedical investigation focusing on developing, implementing, and disseminating clinical therapeutic means, and its approach is a well-defined process involving “bench-to-bedside” laboratory investigations aiming to develop therapeutic means (T1), clinical trials that use the developed means (T2), implementation and dissemination of successful therapeutic strategy (T3), and studies of outcomes (T4) [1,2]. Additionally, “reverse translation” may be used to study observations made in the clinics during T2, using the “bedside-to-bench” approach (Figure 1) [3]. TR is a rapidly progressing discipline first introduced in oncology and spreading to all other biomedical specialties [4], including audiology [5].

Identifying research as “translational” helps others quickly recognize the study’s aims and is critical for preparing grant proposals, developing new studies, or extracting data for systematic reviews or meta-analyses. Additionally, the term “translational audiology” creates a niche in biomedical disciplines and indicates a commitment to convert audiology-related basic findings into clinical approaches, thereby eventually improving the community’s health. An efficient method to identify focused research is Medical Subject Heading (MeSH). MeSH is a stratified vocabulary created and provided by the National Library of Medicine and is used to describe the content of journal articles. Carrying out a search according to the subject content of journal articles rather than to the occurrence of a word or phrase [6] is particularly useful when gaining an understanding of the scope of a field.

Due to the anatomical and biological properties of the inner ear and the auditory pathways, progress in audiology can be achieved by solving clinical problems in clinical [7] or laboratory settings [8], software-simulated situations [9], animal models [10], or explanted tissues [11]. Numerous studies in audiology have a translational character, including research on preventing ototoxicity, EEG-based auditory tests (auditory brainstem responses, ABRs) of noise-exposed animals treated with protective substances, or development of tinnitus- or vertigo-related apps. However, the terms “translational audiology” or “translational research in audiology” seem to be rarely used to label such studies or publications. Hence, our research question was: *‘Can translational research in audiology be identified in the literature based on the use of the phrase “translational audiology” or “translational research in audiology?”’.* Consequently, this scoping review aimed to map translational research in audiology indicated by MeSH terms in the scientific literature, to characterize the primary research goals in the identified research, and recommend future directions.

## 2. Materials and Methods

The search was performed in October 2022 using EndNote 20 without time window restrictions. Two databases (PubMed and Web of Science) were searched using Medical Subject Heading MeSH Terms (Pubmed) and title/keyword/abstract (Web of Science). The keywords used were: “translational audiology”; “translational research” AND “audiology”; and “translational science” AND “audiology”. Inclusion criteria were English as a publication language and primary research; exclusion criteria were review articles, editorials, overviews, and commentaries.

**Figure 2:**
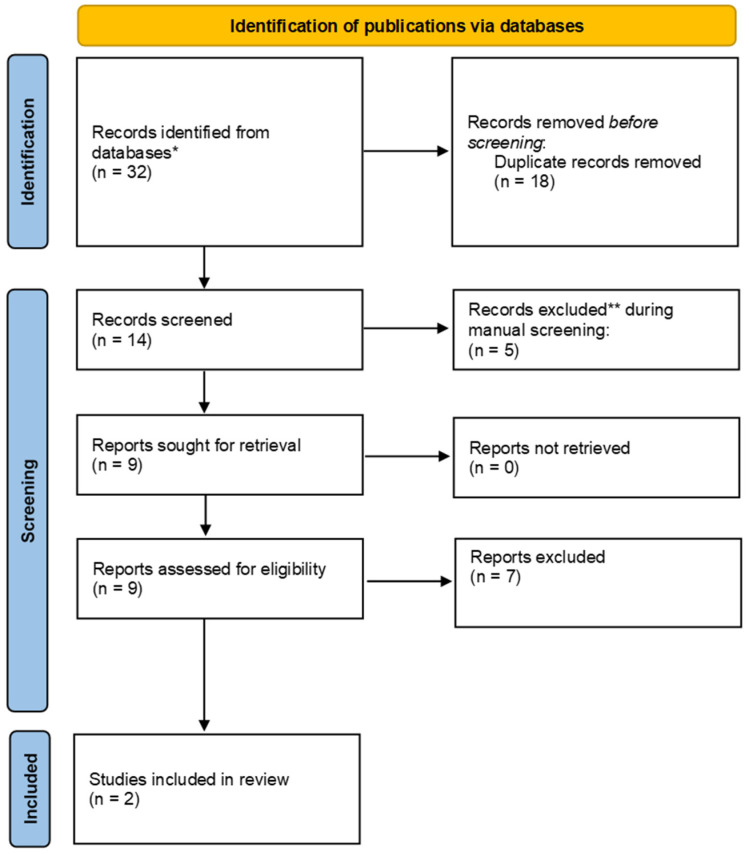
PRISMA flowchart visualizing the search and selection process. * Databases used: PubMed and Web of Science; ** reasons for exclusion: topic outside the scope of the review.

The search retrieved 32 publications (Figure 2). Examination of PubMed with MeSH Terms “translational research” AND MeSH Terms “audiology” returned 3 hits; MeSH Terms “translational research” AND MeSH Terms “audiology”—3 papers; MeSH Terms “translational audiology”—10 hits; and MeSH Terms “translational science” AND MeSH Terms “audiology”—3 hits: 19 publications in total. The search of Web of Science (keyword/title/abstract) using the keyword “translational audiology” retrieved 6 hits; “translational research” AND “audiology”—5 hits; and “translational science” AND “audiology”—2 hits: 13 publications in total. Of these 32 publications, 18 duplicates were identified and removed. Abstracts of the remaining 14 publications were manually screened, and five records were removed because their topics did not involve translational audiology research. The rest of the publications were assessed for eligibility, and seven did not match the inclusion criteria (editorials, reviews, opinions, or overview papers).

Two manuscripts were included in the detailed analysis, and the type of translational research used (T1–T4) and the main study aims were examined.

PubMed and the Web of Science are the most popular sources for publication searches. However, Google Scholar represents another essential search engine that screens not only the journals listed in PubMed or Web of Science but also books. To identify books published on the topic, we performed an additional search in Google Scholar using the terms “translational research” AND “audiology”. That search retrieved 1790 hits; of them, 1220 were books, book chapters, or citations. A manual search identified three books that used the TR term and “audiology” in their titles (see Section 3.2).

## 3. Results

### 3.1. Journal Publications Identified in the Main Search

Two publications were included in this review for the data extraction [12,13]. The goal of the first publication (Kirk, K.I.; Prusick, L.; French, B.; Gotch, C.; Eisenberg, L.S.; Young, N. Assessing Spoken Word Recognition in Children Who Are Deaf or Hard of Hearing: A Translational Approach. Journal of the American Academy of Audiology 2012, 23, 464–475, doi:10.3766/jaaa.23.6.8.) was to “enhance our ability to estimate the real-world listening ability and to predict benefit from sensory aid use in children with varying degrees of hearing loss.” The motivation behind that was a clinical observation that children with a cochlear implant performed poorly on word recognition tests despite their positive performance in real-life situations. That prompted the authors to return to the laboratories and redesign the word recognition tests. In translational research, such an approach represents T2 to T1 methodology (reverse translation). To achieve their goal, the authors have analyzed children’s performance, identified the tests’ shortcomings, and proposed solutions by introducing multimodal sentence tests optimized for children with CI (T3).

The second publication included in this review (Urquiza, R.; Lopez-Garcia, J. A new strategy for development of transducers for middle ear implants. Acta Oto-Laryngol 2015, 135, 135–139, doi:10.3109/00016489.2014.969381.) scrutinized the role of micro-electro-mechanical technology in the design and production of middle ear implants. The authors analyzed the technological and medical situations regarding the available expertise and product roadmap achievement related to T0, T1, and T2 translational steps.

### 3.2. Books Identified in the Additional Search

Using Google Scholar, we identified the following three books:

“Translational Research in Audiology, Neurotology, and the Hearing Sciences”, with ten chapters discussing various aspects of translational research in the field [5].

“Translational Speech-Language Pathology and Audiology: Essays in Honor of Dr. Sadanand Singh”, which contains information on translational research in general and then deals with the translational approach in audiology [14].

“Translational Perspectives in Auditory Neuroscience: Hearing Across the Life Span—Assessment and Disorders”, which introduces the auditory system and discusses translational aspects of audiological diagnostics and therapies [15].

## 4. Discussion

At the beginning of this study, we asked the question: ’Can translational research in audiology be identified in the literature based on the use of the phrase “translational audiology” or “translational research in audiology?”’. The answer to this question is ‘yes’; however, the MeSH term search identified only two peer-reviewed journal publications that met the inclusion criteria. The two manuscripts retrieved during the systematic search of journals had clearly defined translational character and specifically mentioned translational research in audiology. However, no stage of TR (T0–T4) was indicated in these papers and had to be deduced from the body of the text.

The results of the MeSH term search do not represent the actual volume of published translational research in audiology, which is substantially higher. Searches for a specific topic or term (without using TR as a keyword), such as creating or validating audiology-related questionnaires, developing therapeutic strategies for hearing loss, or anti-ototoxic strategies, retrieved hundreds of hits. That, in the light of our study, suggests that the researchers and clinicians are reluctant to use the term “translational audiology” or “translational research” in their published manuscripts. However, this was not the case regarding the books published in the recent decade [5,14,15]. Below, we attempt to analyze the possible reasons behind that reluctance and present incentives to use this terminology.

Translational research aims to implement research findings clinically and ensures that the new therapeutic or monitoring means will reach the appropriate community. The definition of translational research and the steps involved has evolved over the years. Therefore, the first reason for hesitating to use the term “translational research” in audiology could be the multiple meanings awarded to that term over time. These multiple meanings could have been induced by a constant revision of TR’s definition [2,16] and a diverse perception of TR by different scientific fields, including for instance, viewing basic research as non-translational [17]. Supporting this view, in their analysis of publications from various medical disciplines, Krueger et al. showed that many scientific groups use the terminology “translational research” but understand it in various ways. [16]. No study so far has explored the understanding of the term TR among experimental or clinical audiologists.

The second reason behind the reluctant use of TR terminology in publications could be a lack of motivation. Calls for TR-targeted grants could enhance this motivation. For the past four years, the UK-based Royal National Institute for Deaf People and French Fondation Pour l’Audition have held a competition for research grants focused on translational research for hearing loss and tinnitus [18]. In the USA, the National Institute on Deafness and Other Communication Disorders (NIDCD) has offered this kind of grant for almost a decade. Nevertheless, securing a grant related to translational research makes TR more popular as a term but does not assure the use of it while publishing the research outcomes.

Apart from reluctance, the unfamiliarity of the audiological society with TR could also be a reason for the sparse use of that term in published work. Here, it would be suggested to propagate this term among the community of audiologists, for example, during scientific congresses or professional courses.

A final possible reason that could be responsible for the infrequent use of “translational audiology” may be the multidisciplinary approach often used in audiology research [19,20,21]. This approach can sometimes result in publishing with an emphasis on other disciplines, leaving translational audiology overshadowed by, e.g., neurology or otorhinolaryngology.

The obvious challenge is how to enhance the use of the term TR in audiology publications. One encouragement would be to emphasize that applying the term TR could increase the visibility of published studies. Recently, a bibliometric measure of translational science has been proposed to evaluate the translation of basic research into clinics [22]. This method tracks the practical implementation of preclinical research, resulting in the so-called translational score. While it is labor intensive, it focuses on the translational success of a specific manuscript and can also be used to track the practical success of steps T0–T4. An additional incentive would be the possibility of extending the scope of publishing to journals specializing in TR. Another effective solution to increase the popularity and use of the term TR could be an introduction of a well-defined subsection “Translational Audiology” or “Translational Research in Audiology” in specialized journals.

In conclusion, at present, identifying translational research in audiology using MeSH terms is challenging. It could be facilitated by adding TR to the keywords and methods section. Specifying particular translational steps (T0 to T4) could also aid in understanding the research design and lead to additional recognition and more significant credit in the field.

## Data Availability

Not applicable.
